# Single nucleotide polymorphisms within HLA region are associated with the outcomes of unrelated cord blood transplantation

**DOI:** 10.1038/s41598-021-01155-z

**Published:** 2021-11-09

**Authors:** Ding-Ping Chen, Su-Wei Chang, Tang-Her Jaing, Wei-Ting Wang, Fang-Ping Hsu, Ching-Ping Tseng

**Affiliations:** 1 Department of Laboratory Medicine, Chang Gung Memorial Hospital, Taoyuan, 333 Taiwan; 2 Department of Medical Biotechnology and Laboratory Science, College of Medicine, Chang Gung University, Taoyuan, 333 Taoyuan County Taiwan; 3 Graduate Institute of Biomedical Sciences, College of Medicine, Chang Gung University, Taoyuan, 333 Taiwan; 4 Clinical Informatics and Medical Statistics Research Center, College of Medicine, Chang Gung University, Taoyuan, 333 Taiwan; 5 Division of Allergy, Asthma, and Rheumatology, Department of Pediatrics, Chang Gung Memorial Hospital, Taoyuan, 333 Taiwan; 6 Division of Hematology/Oncology, Department of Pediatrics, Chang Gung Children’s Hospital, Chang Gung University, Taoyuan, 333 Taiwan

**Keywords:** Genetic markers, Immunogenetics

## Abstract

Cord blood transplantation (CBT) provides a treatment scheme for hematologic diseases and leukemia in both children and adults. However, adverse reactions and transplantation-related death may still occur in patients receiving CBT even when donor and recipient have fully matched HLA in high-resolution HLA typing analysis. Single nucleotide polymorphisms (SNPs) of HLA-related and unrelated genes are known to associate with disease status of patients with unrelated stem cell transplantation. In this study, the genomic regions ranging from 500 base pairs upstream to 500 base pairs downstream of the eight SNPs that were reported as transplantation determinants by Petersdorf et al. were analyzed to evaluate whether genetic variants were associated with the survival status of patients, and the risk for severe (grades 3–4) graft-versus-host disease (GVHD) or cytomegalovirus (CMV) infection/reactivation. The analyses were performed in the mode of recipient genotype, donor genotype, and recipient-donor mismatching, respectively. By analysis of sixty-five patients and their HLA-matched unrelated donors, we found that five SNPs were associated with patient survival which included the recipient genotype with SNPs of rs107822 in the RING1 gene, and rs2070120, rs17220087 and rs17213693 in the HLA-DOB gene; and the recipient-donor mismatching with SNPs of rs9282369 in HLA-DOA gene, and rs2070120, rs17220087 and rs17213693 in the HLA-DOB gene. Five SNPs were associated with the risk for severe GVHD which included the donor genotype with SNPs of rs213210 and rs2523675; the recipient genotype with SNPs of rs9281491 in the HCP5 gene; and the recipient-donor mismatching with SNPs of rs209130 in the TRIM27 gene, and rs986522 in the COL11A2 gene. Six SNPs were related to the risk for CMV infection/reactivation which included the donor genotype with SNPs of rs435766, rs380924, and rs2523957; and the recipient-donor mismatching with SNPs of rs2070120, rs17220087, and rs17213693 in the HLA-DOB gene; and rs435766 and rs380924 in the MICD gene. This study provides the basis for larger analyses and if the results are confirmed, a way of selecting better unrelated CBT candidate donors.

## Introduction

Human major histocompatibility complex (MHC) is encoded by the human leukocyte antigen (HLA) system located on the chromosome 6. Each individual has a unique HLA expression, providing a means to distinguish own cells from the foreign one in immune system^1^. HLA-matched donor-recipient pair is therefore a primary choice to prevent transplant rejection when receiving allogeneic hematopoietic stem cell transplantation (HSCT)^2^. With the difficulty in finding donors with fully matched HLA stem cells from bone marrow or peripheral blood together with the better transplant tolerance of umbilical cord blood, unrelated cord blood transplantation (CBT) is an alternative scheme for stem cell transplantation.

In the clinical setting, CBT can be used to treat hematologic diseases and cancers (particularly leukemia) in both children and adults^3^. CBT is usually more transplant tolerance allowing one or two allelic mismatches^4,5^. Unfortunately, adverse reactions and transplantation-related death may still occur even when donor and recipient have fully matched HLA as revealed by the high-resolution sequence-based typing^6^. Other vital factors are likely to affect the effectiveness of CBT, in addition to the classical HLA types (HLA-A, -B, -C, -DR, -DP and -DQ).

Single nucleotide polymorphisms (SNPs) of immune-related genes contribute to the onset of disorders and immune reactivity^7^. Previous studies by Petersdorf et al. revealed that the rs2242656 of BAG6, rs986522 of COL11A2, rs2244546 of HCP5, rs2523957 of MICD, rs429916 of HLA-DOA, rs2071479 of HLA-DOB, rs107822 of RING1, and rs209130 of TRIM27 are related to the risk of relapse, survival rate, or graft-versus-host disease (GVHD) in patients receiving bone marrow stem cell transplantation^8^. Specific genotypes of these SNPs in the recipients, donors, or specific pairing of donor-recipient genotype are associated with the outcomes of transplantation. When we analyzed these SNPs in the Taiwanese population, no genotypes in these SNPs are correlated with the risk of disease relapse for patients receiving transplants from bone marrow or umbilical cord blood stem cells. Instead, distinctive sets of SNPs adjacent to these sourced or reference SNPs are associated with disease relapse post-CBT^9^. These SNPs include rs2523675 and rs2518028 at the telomeric end of HCP5 gene, rs2071479 in the intron of the HLA-DOB gene, rs9276982 in the HLA-DOA gene, and rs2523958, rs435766 and rs380924 in the MICD gene.

The studies as abovementioned imply that SNPs relative to the outcomes of stem cell transplantation are likely population- and race-dependent. The association of SNPs in the HLA region with the survival of patients, the severity of GVHD and the occurrence of cytomegalovirus (CMV) infection/reactivation in the Taiwanese population has not yet been explored. These issues were addressed and discussed in this study.

## Results

### Patient characteristics and study design

Patients (n = 65) with hematological disorders (mostly transfusion-dependent thalassemia), or other tumor diseases receiving unrelated CBT from HLA-matched donors were recruited to this study (Table 1). To analyze whether any SNPs within the HLA region are associated with the occurrence of adverse reactions and the survival of patients, the genomic regions 500 bp upstream and downstream of the 8 sourced SNPs (Table 2) for the donor and recipient were amplified by PCR using the forward and reversed primers (Table 3). PCR amplicons were sequenced and analyzed to investigate whether there were candidate SNPs related to the survival of patients and the occurrence of adverse reactions. Three different modes including donor genotype analysis, recipient genotype analysis, and mismatch between donor-recipient pair (defined by having a specific combination of SNP alleles between the donor and recipient) were performed to correlate specific SNPs with the clinical outcomes post-transplantation.

**Table 1:** Characteristics of patients receiving unrelated CBT.

	Number of patient (%) or median (range)	
Number of patients	65	
Median age in years at transplantation (range)	5 years old (27 days-15 years old)	
Male: Female	39 (60%): 26 (40%)	
**Diagnosis**		
Transfusion-dependent thalassemia	26	40.0%
Severe aplastic anemia	5	7.7%
Fanconi anemia	3	4.6%
ALL	7	10.8%
AML	3	4.6%
CML	1	1.5%
Inheritable disease	14	21.5%
(chronic primary granulomatous disease, X-linked severe combined immunodeficiency, Wiskott-Aldrich Syndrome, malignant osteopetrosis)		
Tumor disease	6	9.2%
(neuroblastoma, retroperitoneal neuroblastoma, malignant tumor)		
**Matching at antigen-level for HLA-A and -B and allele-level for HLA-DRB1**		
Fully matched	15	23.1%
One mismatch	27	41.5%
Two mismatches	23	35.4%
**ABO compatibility**		
Full match	31	47.7%
Minor mismatch	18	27.7%
Major mismatch	16	24.6%
Overall survival	50	76.9%
**GVHD**		
Non-GVHD	11	16.9%
Grade 1–2	27	41.5%
Grade 3–4	14	21.5%
Chronic	13	20.0%
**CMV infection/reactivation**		
Negative	45	69%
Positive	20	31%

**Table 2:** The SNPs flanking the 8 sourced SNPs under analysis in this study.

Gene	Sourced SNP		SNP under analysis	
BAG6	**rs2242656**	rs3130048	rs2844464	rs2242656
COL11A2	**rs986522**	rs77011831	rs986522	rs115641163
		rs986521	rs2229784	
HCP5	**rs2244546**	rs9281491	rs2244546	rs4713466
		rs2523676	rs2523675	rs2518028
		rs1414315		
MICD	**rs2523957**	rs435766	rs380924	rs1264813
		rs2523960	rs2523959	rs2523958
		rs2523957		
HLA-DOA	**rs429916**	rs9276982	rs71565361	rs79327197
		rs151190962	rs9282369	
HLA-DOB	**rs2071479**	rs11244	rs2070120	rs56150445
		rs41258084	rs17220087	rs2071479
		rs17213693		
RING1	**rs107822**	rs107822	rs213210	
TRIM27	**rs209130**	rs209132	rs209131	rs209130
		rs1536215	rs139791445

**Table 3:** The primer sequences for amplifying genomic region flanking the sourced SNPs.

Gene	Primer sequences
BAG6	F: 5'-ATTCATTCAGGGGCACAAGGGG-3' R: 5'-GCGGAGGTTGAAGAGAATAGAAGC-3'
COL11A2	F: 5'-TGTCCCTCACCTTGGCTCCCTT-3' R: 5'-AATTCCTCTCTCCCTAGGGAT-3'
HCP5	F: 5'-GGGCAACTAAGTCAGGTCTAG-3' R: 5'-TCTGCAGGTCTCATGGAGAG-3'
HLA-A	F: 5'-TTCCAAGTGAGGAACTCAGACC-3' R: 5'-AAGATGCACTGATCCTCCCT-3'
HLA-DOA	F: 5'-CAACAACGTAAAGCTAACGTCTGTG-3' R: 5'-GCACCACTCTTAGTTATGTATAGG-3'
HLA-DOB	F: 5'-TCTTCTGAAGACTGTGGAGACTGC-3' R: 5'-TCCCATAGGAGCTCAGTCTGAAT-3'
RING1	F: 5'-TAATCGACTCTGGCGCCCACAT-3' R: 5'-AACAACCTTAGCCTCGGTTCCCTT-3'
TRIM27	F: 5'-AGTCGGGATTACAGAAATGCACC-3' R: 5'-GCAGGACATTTGAAGGTAACC-3'

*F* forward primer, *R* reversed primer.

### Association of SNPs with adverse reactions and survival post-CBT

Five SNPs were associated with the occurrence of adverse reactions post-CBT in the donor genotype analysis (Table 4). Three SNPs located in the MICD gene were related to the risk for CMV infection/reactivation. When the donor had the AA genotype in the rs435766, the recipient had a higher risk for CMV infection/reactivation (p = 0.031, OR = 4.667, 95% CI 1.251–17.409). Moreover, the rs380924 and the rs2523957 with GG genotype were also associated with a higher risk for CMV infection/reactivation (p = 0.031, OR = 0.214, 95% CI = 0.057–0.799). On the other hand, two SNPs were related to the occurrence of severe GVHD (grades 3–4), including rs213210 located on the upstream of RING1 gene (p = 0.028) and rs2523675 located on 2.4 kb telomeric of HCP5 gene (p = 0.016). No SNP had statistical correlation with the survival of recipient in the donor genotype analysis.

**Table 4:** The SNPs associated with the outcomes of unrelated CBT in donor genotype analysis.

SNP	Gene	Chromosome position (bp)	Source	Outcome/ Status	Donor genotype frequency (%)			Test	*p*-value	OR (95% CI)
rs435766	MICD	Chr6: 29,939,852	rs2523957	CMV	AA	AG	GG	Recessive	**0.031**	4.667 (1.251–17.409)
				Yes	7 (58.3)	6 (17.6)	6 (33.3)			
				No	5 (41.7)	28 (82.4)	12 (66.7)			
rs380924	MICD	Chr6: 29,939,885	rs2523957	CMV	AA	AG	GG	Recessive	**0.031**	0.214 (0.057–0.799)
				Yes	6 (33.3)	6 (17.6)	7 (58.3)			
				No	12 (66.7)	28 (82.4)	5 (41.7)			
rs2523957	MICD	Chr6: 29,940,260	rs2523957	CMV	AA	AG	GG	Recessive	**0.031**	0.214 (0.057–0.799)
				Yes	6 (33.3)	6 (17.6)	7 (58.3)			
				No	12 (66.7)	28 (82.4)	5 (41.7)			
rs213210	RING1	Chr6: 33,175,824	rs107822	GVHD = 3 & 4	AA	AG	GG	Recessive	**0.028**	0.217 (0.058–0.817)
				Yes	6 (42.9)	6 (17.6)	1 (6.3)			
				No	8 (57.1)	28 (82.4)	15 (93.8)			
rs2523675	HCP5	Chr6: 31,436,032	rs2244546	GVHD = 3 & 4	AA	AG	GG	Dominant	**0.016**	5.20 (1.430–18.912)
				Yes	4 (25.0)	1 (3.6)	8 (40.0)			
				No	12(75.0)	27 (96.4)	12 (60.0)			

Dominant: dominant model (AA vs. Aa + aa); Recessive: recessive model (AA + Aa vs. aa), in which “A” was defined as a higher frequency allele and the lower was “a”.

Data were analyzed by Chi-square test or Fisher's exact test.

In the recipient genotype analysis, six SNPs were related to the survival of recipients or the occurrence of severe GVHD (grades 3–4) (Table 5). The (CC + CT) alleles in rs107822 located 2.0 kb upstream of RING1 gene was associated with a higher survival for recipients (p = 0.017, OR = 4.909, and 95% CI = 1.229–19.606). Three SNPs (rs2070120, rs17220087, and rs17213693) located in the intron or 3'-UTR of HLA-DOB were associated with the survival of recipients based on the dominant model of analysis (p = 0.027, OR = 0.178, and 95% CI = 0.040–0.783). On the other hand, two SNPs located on 2.2 kb or 2.3 kb telomeric of HCP5 gene were related to the occurrence of severe GVHD (grades 3–4). Patients with the AA genotype in rs9281491 had higher risk for severe GVHD (p = 0.013, OR = 10.889, and 95% CI = 1.729–68.576), while the patients with T-allele in rs4713466 had lower risk for severe GVHD comparing to CC genotype (p = 0.013, OR = 0.092, 95% CI = 0.015–0.578). No SNP was statistically correlated with CMV infection/reactivation in the recipient genotype analysis.

**Table 5:** The SNPs associated with the outcomes of unrelated CBT in recipient genotype analysis.

SNP	Gene	Chromosome position (bp)	Source	Outcome /status	Recipient genotype frequency (%)			Test	*p*-value	OR (95% CI)
rs107822	RING1	Chr6: 33,175,575	rs107822	Survival	CC	CT	TT	Dominant	**0.017**	4.909 (1.229–19.606)
				Yes	5 (100.0)	22 (88.0)	22 (64.7)			
				No	0 (0)	3 (12.0)	12 (35.3)			
rs2070120	HLA-DOB	Chr6: 32,780,914	rs2071479	Survival	AA	AG	GG	Dominant	**0.027**	0.178 (0.040–0.783)
				Yes	0 (0)	4 (50.0)	45 (81.8)			
				No	1 (100.0)	4 (50.0)	10 (18.2)			
rs17220087	HLA-DOB	Chr6: 32,781,076	rs2071479	Survival	AA	AC	CC	Dominant	**0.027**	0.178 (0.040–0.783)
				Yes	0 (0)	4 (50.0)	45 (81.8)			
				No	1 (100.0)	4 (50.0)	10 (18.2)			
rs17213693	HLA-DOB	Chr6: 32,781,121	rs2071479	Survival	CC	CG	GG	Dominant	**0.027**	0.178 (0.040–0.783)
				Yes	0 (0)	4 (50.0)	45 (81.8)			
				No	1 (100.0)	4 (50.0)	10 (18.2)			
rs9281491	HCP5	Chr6: 31,435,815:	rs2244546	GVHD = 3 & 4	AA	A-	–	Recessive	**0.013**	10.889 (1.729–68.576)
		31,435,816		Yes	4 (66.7)	6 (21.4)	3 (10.0)			
				No	2 (33.3)	22 (78.6)	27 (90.0)			
rs4713466	HCP5	Chr6: 31,435,869	rs2244546	GVHD = 3 & 4	CC	CT	TT	Dominant	**0.013**	0.092(0.015–0.578)
				Yes	12 (27.9)	1 (5.9)	0 (0)			
				No	31 (72.1)	16 (94.1)	4 (100.0)			

Dominant: dominant model (AA vs. Aa + aa); Recessive: recessive model (AA + Aa vs. aa), in which “A” was defined as a higher frequency allele and the lower was “a”. Data were analyzed by Chi-square test or Fisher's exact test.

For the mismatch between donor-recipient pair genotype analysis, nine SNPs were related to the survival of patients or the occurrence of adverse reactions (Table 6). Among the nine SNPs, four SNPs were related to the survival of patients, six SNPs were associated with CMV infection/reactivation, and two SNPs were correlated with the occurrence of severe GVHD. Three SNPs (rs2070120, rs17220087, and rs17213693) were related to more than one outcome post-CBT. When the genotype of rs9282369 located 2.3 kb upstream of HLA-DOA gene was matched between donor and recipient, the recipients had a less favorable survival (p = 0.005, OR = 0.181, 95% CI = 0.052–0.628). When the genotypes of rs2070120, rs17220087, and rs17213693 located in the intron or 3’-UTR of HLA-DOB gene were not matched between the donors and recipients, the recipients had a less favorable survival (p = 0.014, OR = 7.667, 95% CI = 1.569–37.458). The same three SNPs in the HLA-DOB gene (rs2070120, rs17220087, and rs17213693) were also associated with the risk for CMV infection/reactivation. When the genotype of these SNPs was matched between donor and recipient, the recipients had a lower risk for CMV infection/reactivation (p = 0.044, OR = 0.200, 95% CI = 0.042–0.946). However, when the genotypes were matched between donor and recipient for the three SNPs located in the MICD gene, the recipients had a higher risk for CMV infection/reactivation (rs435766, p = 0.009; rs380924, p = 0.014; and rs1264813, p = 0.042). When the genotype of rs209130 located on 3.0 kb downstream of TRIM27 gene was matched between donor and recipient, the recipients were more likely to develop severe GVHD (p = 0.043, OR = 3.625, 95% CI = 0.996–13.194). On the other hand, when the genotype of rs986522 located in the intron of COL11A2 gene was matched between donor and recipient, the recipients were less likely to develop severe GVHD (p = 0.021, OR = 0.210, 95% CI = 0.051–0.856).

**Table 6:** The SNPs associated with the outcomes of unrelated CBT in donor-recipient pairs analysis.

SNP	Gene	Chromosome position (bp)	Source	Outcome/status	Genotypes between the Donor-Recipient Pairs (%)		*p*-value	OR (95% CI.)
rs9282369	HLA-DOA	Chr6: 32,978,788:	rs429916	Survival	Matched	Not matched	**0.005**	0.181 (0.052–0.628)
		32,978,789		Yes	13 (56.5)	36 (87.8)		
				No	10 (43.5)	5 (12.2)		
rs2070120	HLA-DOB	Chr6: 32,780,914	rs2071479	Survival	Matched	Not matched	**0.014**	7.667 (1.569–37.458)
				Yes	46 (82.1)	3 (37.5)		
				No	10 (17.9)	5 (62.5)		
rs17220087	HLA-DOB	Chr6: 32,781,076	rs2071479	Survival	Matched	Not matched	**0.014**	7.667 (1.569–37.458)
				Yes	46 (82.1)	3 (37.5)		
				No	10 (17.9)	5 (62.5)		
rs17213693	HLA-DOB	Chr6: 32,781,121	rs2071479	Survival	Matched	Not matched	**0.014**	7.667 (1.569–37.458)
				Yes	46 (82.1)	3 (37.5)		
				No	10 (17.9)	5 (62.5)		
rs2070120	HLA-DOB	Chr6: 32,780,914	rs2071479	CMV	Matched	Not matched	**0.044**	0.200 (0.042–0.946)
				Yes	14 (25.0)	5 (62.5)		
				No	42 (75.0)	3 (37.5)		
rs17220087	HLA-DOB	Chr6: 32,781,076	rs2071479	CMV	Matched	Not matched	**0.044**	0.200 (0.042–0.946)
				Yes	14 (25.0)	5 (62.5)		
				No	42 (75.0)	3 (37.5)		
rs17213693	HLA-DOB	Chr6: 32,781,121	rs2071479	CMV	Matched	Not matched	**0.044**	0.200 (0.042–0.946)
				Yes	14 (25.0)	5 (62.5)		
				No	42 (75.0)	3 (37.5)		
rs435766	MICD	Chr6: 29,939,852	rs2523957	CMV	Matched	Not matched	**0.009**	6.800 (1.402–32.972)
				Yes	17 (40.5)	2 (9.1)		
				No	25 (59.5)	20 (90.9)		
rs380924	MICD	Chr6: 29,939,885	rs2523957	CMV	Matched	Not matched	**0.014**	6.212 (1.279–30.159)
				Yes	17 (39.5)	2 (9.5)		
				No	26 (60.5)	19 (90.5)		
rs1264813	MICD	Chr6: 29,939,900	rs2523957	CMV	Matched	Not matched	**0.042**	4.690 (0.959–22.933)
				Yes	17 (37.0)	2 (11.1)		
				No	29 (63.0)	16 (88.9)		
rs209130	TRIM27	Chr6: 28,867,800	rs209130	GVHD = 3 & 4	Matched	Not matched	**0.043**	3.625 (0.996–13.194)
				Yes	10 (71.4)	4 (28.6)		
				No	20 (40.8)	29 (59.2)		
rs986522	COL11A2	Chr6: 33,135,962	rs986522	GVHD = 3 & 4	Matched	Not matched	**0.021**	0.210 (0.051–0.856)
				Yes	3 (23.1)	10 (76.9)		
				No	30 (58.8)	21 (41.2)		

### Linkage disequilibrium analysis

Pair-wise linkage disequilibrium (LD) analysis of the forty-one SNPs was performed to determine whether there was non-random association of alleles at two or more loci in a general population. D’ was defined as the normalized standard measurement of LD by comparing the observed and expected frequencies of one haplotype comprised by alleles at different loci. Out data revealed that the seven SNPs in the MICD gene were all in high LD (pair-wise D’ measures ranged from 0.88 to 1) and formed a haplotype block, implying that these SNPs formed a genetic linkage (Figure S1). The other three haplotype blocks were each comprised of two SNPs located in the RING1, BAG6 and HCP5 genes, respectively.

### Temporal effects of outcomes-related SNPs in CBT

**Figure 1:**
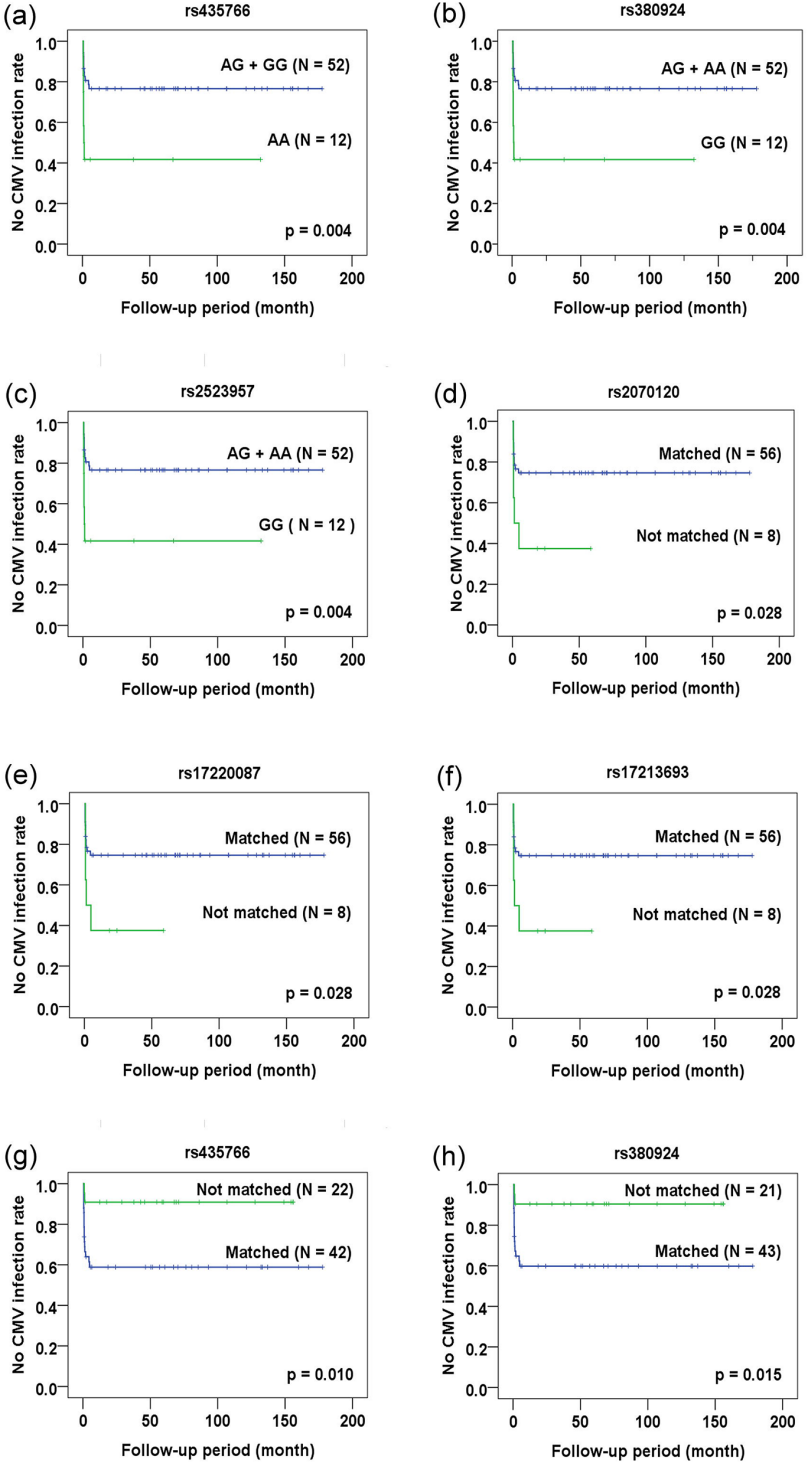
Kaplan–Meier analysis of CMV infection/reactivation-free duration. (**a**–**h**) CMV-related SNPs as revealed by end-point analysis were subject to Kaplan–Meier analysis. The CMV infection/reactivation-free status for recipients with the indicated genotype (panels **a**–**c**) or matched vs. not matched status for the donor and recipient genotype (panels **d**–**h**) was plotted against the follow-up period (month) after transplantation.

**Figure 2:**
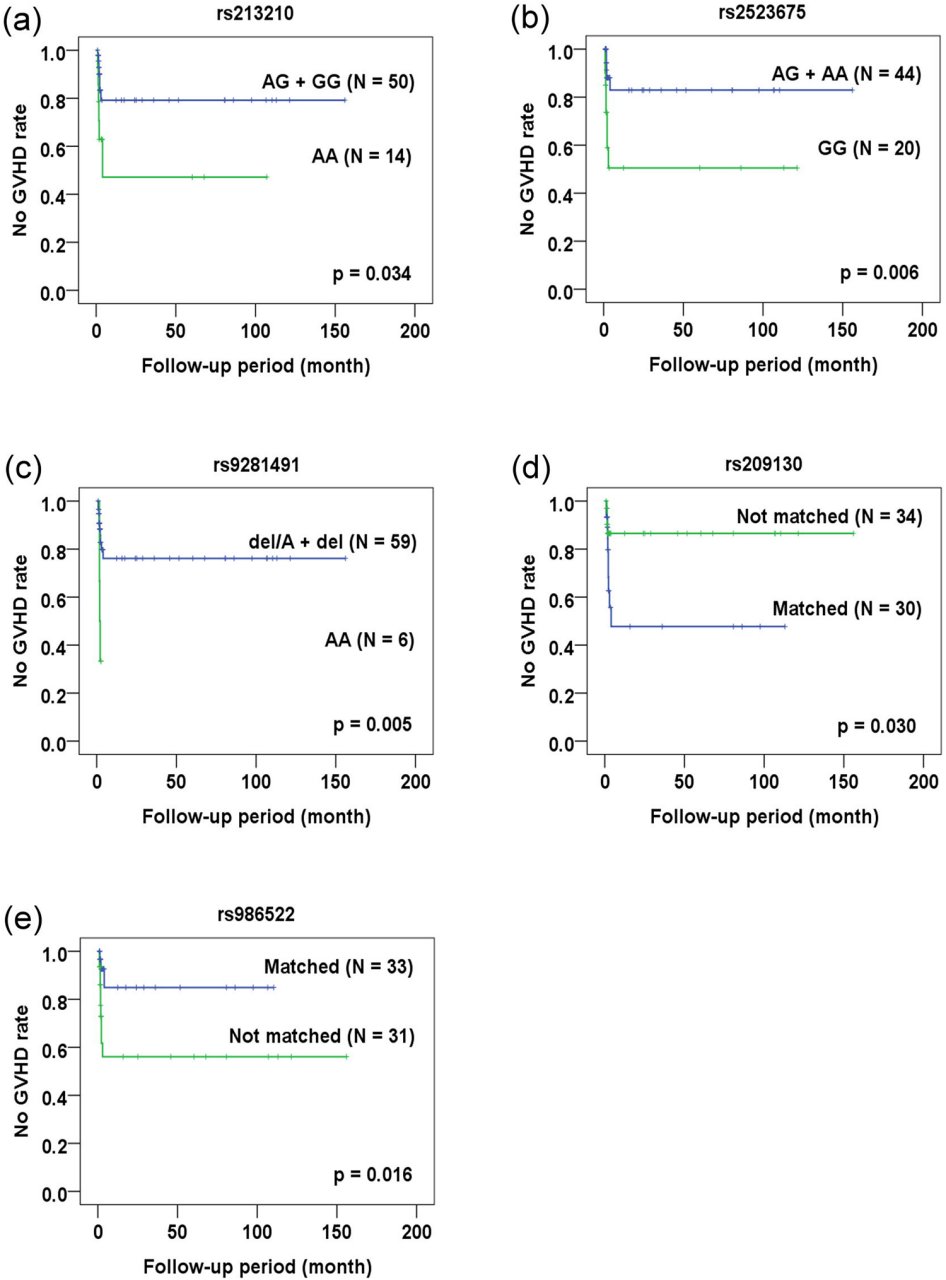
Kaplan–Meier analysis of severe GVHD-free duration. (**a**–**e**) Severe GVHD-related SNPs as revealed by end-point analysis were subject to Kaplan–Meier analysis. The severe GVHD-free status for the SNPs with the indicated genotypes (panels **a**–**c**) or matched vs. not matched status for the donor and recipient genotypes (panels **d**,**e**) was plotted against the follow-up period (month) after transplantation.

**Figure 3:**
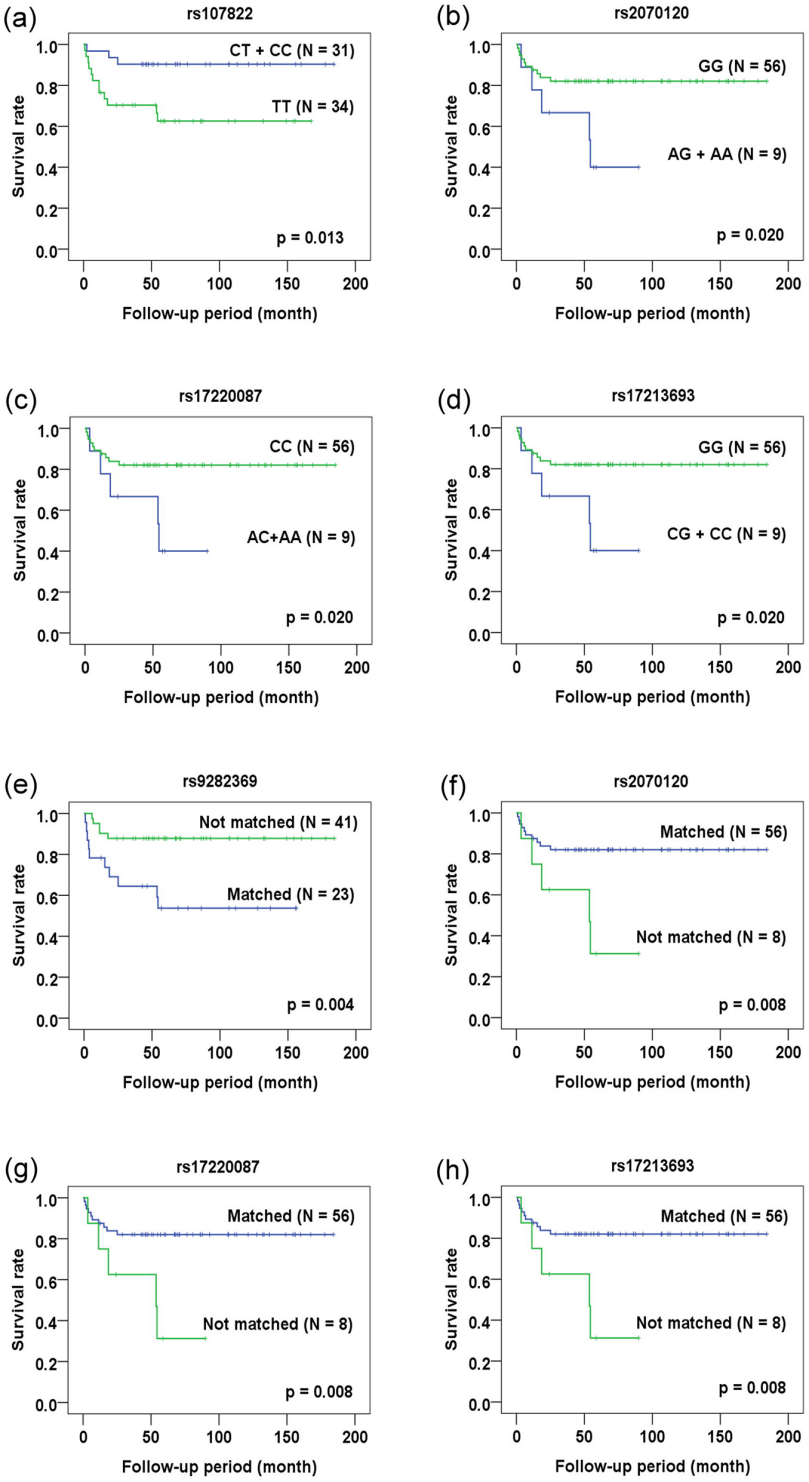
Kaplan–Meier analysis of overall survival. (**a**–**h**) The survival-related SNPs as revealed by end-point analysis were subject to Kaplan–Meier analysis. The overall survival status for recipients with the indicated genotypes (panels **a**–**d**) or matched vs not matched status for the donor and recipient genotype was plotted against the follow-up period (month) after transplantation.

In addition to endpoint study, the association of outcome-related SNPs with the occurrence of adverse reactions and the survival of recipients was analyzed and confirmed by counting on the time-effect. Event-free duration was defined as the time from patients receiving CBT to the occurrence of adverse reactions or death. By Kaplan–Meier analysis, the association of CMV-related SNPs with CMV infection/reactivation (Fig. 1) was confirmed (in donor genotype analysis: rs435766, p = 0.004; rs380924, p = 0.004; and rs2523957, p = 0.004; in mismatch between donor-recipient pair genotype analysis: rs2070120, p = 0.028; rs17220087, p = 0.028; rs17213693, p = 0.028; rs435766, p = 0.010; and rs380924, p = 0.015). Only the rs1264813 of MICD gene failed to demonstrate its association with CMV reactivation (p > 0.05). All GVHD-related SNPs except rs4713466 (Fig. 2) were associated with the occurrence of severe GVHD when time-dependent effect was included in the analysis (in donor genotype analysis: rs213210, p = 0.034; and rs2523675, p = 0.006; in recipient genotype analysis: rs9281491, p = 0.005; in mismatch between donor-recipient pair genotype analysis: rs209130, p = 0.030; and rs986522, p = 0.016). All survival-related SNPs (Fig. 3) were related to the overall survival of patients when counting on time-dependent effect (in recipient genotype analysis: rs107822, p = 0.013; rs2070120, p = 0.020; rs17220087, p = 0.020; and rs17213693, p = 0.020; in mismatched between donor-recipient pair genotype analysis: rs9282369, p = 0.004; rs2070120, p = 0.008; rs17220087, p = 0.008; and rs17213693, p = 0.08). These data confirm the association of specific SNP genotypes with the occurrence of adverse reactions and the survival of patients.

## Discussion

CMV infection/reactivation, severe GVHD, and relapse are usually occurred after transplantation. In our previous study, we revealed the association of four HLA-related SNPs in the donor group (rs2523675, rs2518028, rs2071479, and rs2523958) and three HLA-related SNPs in the recipient group (rs9276982, rs435766, and rs380924) with disease relapse in CBT cases^9^. In present study, we expanded the analysis and found that five SNPs in the HLA regions were associated with the survival of patients (rs107822, rs2070120, rs17220087, rs17213693, and rs9282369), six SNPs were associated with CMV infection/ reactivation (rs435766, rs380924, rs2523957, rs2070120, rs17220087, and rs17213693), and five SNPs were associated with the development of severe GVHD (rs213210, rs2523675, rs9281491, rs209130, and rs986522). Moreover, the SNPs of rs2070120, rs17220087, and rs17213693 located in the HLA-DOB genes correlate with more than one outcome post-CBT. These outcomes-related SNPs were confirmed by both end-point and Kaplan–Meier analyses. The outcome-related SNPs as revealed in this study represent novel molecular markers related to the occurrence of adverse reactions and survival of patients receiving unrelated CBT.

The outcomes-related SNPs are mainly located in or adjacent to the MICD, RING1, HCP5, HLA-DOA, HLA-DOB, TRIM27, and COL11A2 genes, implying that these SNPs may be related to the function or expression of the abovementioned genes. MICD gene was considered as a pseudogene located within the MHC class I region. However, recent studies indicate that the start sequence of the HLA complex group 9 (HCG9) corresponds with part of the MICD sequence^10^. The effectiveness of transplantation and the mechanism of disease related to the SNPs in the MICD gene are not clear. Instead of being recognized as a pseudogene, MICD is likely to play a role in basic physiology and disease progression^11,12^. This is consistent with the notion that pseudogenes may encode protein under certain circumstances^13^. The possibility that the MICD has an unknown functional impact on the outcome of CBT cannot be ruled out. In this regard, the SNP of rs380924 adjacent to MICD gene that we showed to associate with CMV infection/reactivation has been found to relate to the risk for psoriasis^14^. The findings of this and previous studies implicate that the SNPs of rs435766, rs380924, and rs2523957 in the MICD gene are crucial to the susceptibility of various diseases including CMV infection/reactivation.

Our data revealed that three SNPs (rs2070120, rs17220087, and rs17213693) located in the intron or 3’-UTR of HLA-DOB regions are associated with the survival of patients in the recipient and donor-recipient matching groups, as well as CMV infection/reactivation in the donor-recipient matching group. The rs9282369 in the upstream promoter region of HLA-DOA, a paralogue of HLA-DOB, is also associated with the survival of patients in donor-recipient matching group. These data imply that both HLA-DOA and -DOB are important in modulating the effectiveness of transplantation. HLA-DO belongs to the HLA class II homologous genes. Both HLA-DOA and -DOB are heterodimer composed of alpha chain and beta chain. Compared with the typical HLA class II molecules, HLA-DO has limited number of polymorphisms^15,16^. HLA-DO interferes MHC-bound epitopes presentation by mediating the function of HLA-DM^17^. The three SNPs in the region of HLA-DOB gene may cause excessive HLA-DOB expression leading to a less effective antigen presentation and lower immune response. On the other hand, DNA polymorphism in the promoter region is known to regulate gene expression^18^. It is worthy to investigate whether the rs9282369 located in the HLA-DOA promoter region regulates HLA-DOA expression leading to the change of patient survival post-CBT.

For the GVHD-related SNPs, the polymorphism of rs213210 was related to GVHD in the donor genotype analysis. The rs213210 is considered not only as the SNP in the promoter region of RING1 gene, but also as the SNP within the pre-miR-219 gene. Wu et al*.* indicated that this gene was related to gastric cancer and showed that the nucleotide changes from T to C in rs213210 increase the expression of miR-219-1^19^. Moreover, there were specific miRNAs that could be served as a biomarker for acute GVHD^20^. Based on our results, the relationship between miR-219 and acute GVHD can be further explored in the future. There are 2 SNPs (rs2523675 and rs9281491) at the telomeric of HCP5 gene related to the occurrence of severe GVHD. HCP5 gene expression is regulated by IFN-γ and MHC class I genes^21^. IFN-γ is an important cytokine in proliferation and differentiation of T cells^22^. It is likely that patients with different SNP genotypes response differently to IFN-γ and elicit differential effects on preventing acute GVHD. The rs209130 located downstream of TRIM27 and the rs986522 in the intron of COLI11A2 gene were associated with GVHD in donor-recipient matching group. The association of rs209130 with severe GVHD may be caused by the abnormal regulation of CD4^+^ T cells^23^ leading to the patient's own tissue cells attacked by over-reconstructed CD4^+^ T cells^24^. On the other hand, the strong linkage disequilibrium^25^ of the SNPs in COL11A2 and HLA-DP and the risk of GVHD related to HLA-DPB1 mismatching in unrelated HSCT^26^ may provide an explanation for the association of rs986522 with GVHD.

We noted that the number of cases in this study is limited, although the specimens were collected over a period of more than ten years with an average of five to ten cases of CBT per year available in our hospital. This represents a limitation of this study. Increasing the enrolled number of donor-recipient pairs to this study or analysis of another large cohort may further validate and confirm the association of these SNPs with the outcomes of unrelated CBT. We also noted that 23% of patients had fully matched CB grafts, and the remaining had one or two mismatches. The cord blood unit up to two allele mismatches between the donor and recipient can be used without an increased risk of allograft rejection in most CBT cases^27^. With the limited number of donor-recipient pairs, it is not feasible to perform further analysis and remains unclear whether the number of HLA mismatch has any impact on SNP association with clinical outcomes. A cohort comprising large number of patients with fully-matched HLA, and one or two mismatched HLA to the donors is required to address this issue. Moreover, multiple factors likely contribute to the occurrence of adverse reactions and the survival of patients. Multivariate analyses including more variables are worthy to perform in the future study.

In conclusion, this study provides a foundation for creating a screening panel of SNPs for seeking a suitable donor before receiving CBT. The successful rate of CBT can be improved by selection of appropriate SNPs in donor genotype, recipient genotype, or mismatch between donor-recipient pair to avoid the occurrence of adverse reactions and improve the survival of patients. Because the genes which carry these SNPs are related to immunological functions or the susceptibility to the immunological disorders, clarifying the effects of these SNPs on the biological functions of these genes and the underlying mechanism should further provide explanations for the association of these SNPs with the outcomes of unrelated CBT.

## Materials and methods

### Patients and HLA typing

This study was approved by the Institutional Review Board of Chang Gung Memorial Hospital with the approval ID of 102-4949B. All methods were performed in accordance with the relevant guidelines and regulations. A total of sixty-five donor-recipient pairs undergoing unrelated CBTs were recruited at Chang Gung Memorial Hospital. The clinical characteristics and indicated diseases were shown in Table 1. All participants provided written informed consents to participate in this study.

HLA typing of HLA-A, -C, -B, -DRB1, -DQB1 alleles for donors and recipients were implemented prior to the transplantation. First, the method of LABType SSO Typing Test (Thermo Fisher, Waltham, MA) was employed along with sequence-specific oligonucleotide probes. Second, the SeCore kit (Thermo Fisher, Waltham, MA) was used for high-resolution HLA typing to obtain more detailed allele information. Finally, the MicroSSP Allele Specific Typing Tray (Thermo Fisher, Waltham, MA) was used to resolve ambiguous alleles of the SeCore typing with sequence-specific primers.

### Definition

The diagnosis of CMV infection/reactivation was based on the detection of CMV antigen or DNA in the peripheral blood of patient after transplantation. CMV antigen in white blood cells was determined by CMV Antigenemia Assay (MONOFLUO™, Bio-Rad). This method can detect CMV viremia earlier and more sensitive than traditional virus culture or shell vial. The test was considered positive when more than 2 polymorphonuclear leukocytes (PMN) were positive for CMV antigen in a total of 50,000 PMN. CMV DNA Quantitative Amplification test is a real-time quantitative PCR assay (COBAS® AmpliPrep/COBAS® TaqMan® CMV Test, Roche). The nucleic acid test was considered positive when the Ct < 37. These two assays can assist clinicians in monitoring the status of CMV infection or reactivation before and after transplantation.

GVHD was defined in accord with the National Institutes of Health (NIH) Consensus. Acute GVHD (aGVHD) was defined as the syndrome occurred with 100 days after transplantation. Otherwise, it was classified as chronic GVHD. According to the clinical characteristics of organs, aGVHD was divided into 4 grades. grade 1: only mild symptoms on the skin; grade 2: slightly serious symptoms on the skin and the mild symptoms on the liver and gastrointestinal tract; grade 3: symptoms on more than half of the skin and severe symptoms on the liver and gastrointestinal tract; grade 4: organs are not able to function properly^28^. In our analysis, GVHD was classified as grades 3–4. vs. else, because the grades 3–4 of GVHD was considered as severe GVHD. Patients who were still alive at the end of this study were classified as survival. Death was defined when patients were died by any reason after receiving transplantation. The event-free duration was defined as the duration from transplantation to the event (CMV infection/reactivation, GVHD grades 3–4, or death) occurred.

### Selection of SNPs

The 8 SNPs (rs2244546, rs986522, rs2244546, rs2523957, rs429916, rs2071479, rs107822, and rs209130) within the HLA region have been shown to associate with the risk of mortality, disease-free survival, transplant-related mortality, relapse and GVHD in patients with HSCT^8^. These SNPs were selected as the sourced SNPs in this study.

The genomic regions ranging from 500 bp upstream to 500 bp downstream of these 8 sourced SNPs were amplified and sequenced to search for candidate SNPs that were associated with the adverse reactions and survival of patients in unrelated CBT. A total of 41 SNPs was present in these regions (Table 2). The association between these SNPs and the risk for the occurrence of adverse reactions or the survival of patients were analyzed and conferred by donor SNP (mode of donor genotype analysis), recipient SNP (mode of recipient genotype analysis) or mismatched of donor-recipient pair SNP (mode of mismatch between donor-recipient pair, defined by having a specific combination of different SNP alleles between the donor and recipient) as described previously^14^.

### PCR and sequencing

The genomic DNA from the peripheral blood of recipients was isolated by using the QIAamp DNA Blood mini Kit (Qiagen, Valencia, CA). The genomic DNA from the donors was extracted from a small segment of blood in the infusion tube connected to the blood bag by using the same DNA extraction method. A total of 8 different primer pairs (Table 3) were used to amplify the DNA fragments that covered from 500 bp upstream to 500 bp downstream of the 8 sourced SNPs by PCR as described previously^9^. Briefly, PCR was performed in a reaction volume of 50 μl containing 1X reaction buffer, 10 nmol of dNTPs, 6 pmol of forward and reverse primers, 300 ng of genomic DNA, and 1 μl of Pfu Turbo Hotstart DNA Polymerase (Agilent, Santa Clara, CA). The PCR program was 4 min at 94 °C for 1 cycle, 30 s at 94 °C, 30 s at 58 °C, and 45 s at 72 °C for 30 cycles, and 10 min at 72 °C for 1 cycle. Subsequently, 5 μl of PCR products were fractionated on a 2% agarose gel and visualized by ethidium bromide staining. The remaining PCR product was subject to direct sequencing using the Big Dye Terminator Cycle Sequencing kit (Thermo Fisher, Waltham, MA) and an ABI PRISM Genetic Analyzer (Thermo Fisher, Waltham, MA) according to the manufacturer’s instructions.

### Statistical analysis

The Hardy–Weinberg equilibrium (HWE) test was performed in control group to examine the quality of experiments for the tested SNPs. SNPs that violated the HWE were eliminated from analysis. The frequencies of allele and genotype were computed and compared between each of the three dichotomized outcome groups: (1) survived vs. deceased; (2) GVHD grades 3–4 vs. else; and (3) having CMV infection/reaction vs. not. The Cochran-Armitage test for trend was performed to evaluate the additive effect of risk alleles that each SNP had on the outcome. The correlation of specific SNP genotypes with different outcomes was investigated by genotypic test. The association between each outcomes and mismatch status of SNP genotypes in donor-recipient matching group was examined using the chi-square and Fisher’s exact tests under three types of models: additive (AA vs. Aa vs. aa), dominant (AA vs. Aa + aa), and recessive (AA + Aa vs. aa) ^29^. Kaplan–Meier analysis and log-rank test were performed by using SPSS ver 17.0. The measurements of pair-wise linkage disequilibrium (LD) D’ and r^2^ for the SNPs in donor group, recipient group, and donor-recipient matching group which refers to the non-random association of alleles at two or more loci in a general population were determined by using HaploView 4.2 (https://www.broadinstitute.org/haploview/haploview). ^30^.

## Supplementary Information

Supplementary Information.
