# Supplementary material for: Single nucleotide polymorphisms within HLA region are associated with the outcomes of unrelated cord blood transplantation
Source: Sci Rep. 2021 Nov 9;11:21925. doi: 10.1038/s41598-021-01155-z (PMC8578435; doi:10.1038/s41598-021-01155-z)
Supplement: Supplementary file 1 — Supplementary Information. [file 41598_2021_1155_MOESM1_ESM.doc]

Single nucleotide polymorphisms within HLA region are associated with the outcomes of unrelated cord blood transplantation

Ding-Ping Chen^1,2,3†^, Su-Wei Chang^4,5†^, Tang-Her Jaing^6†^, Wei-Ting Wang^1^, Fang-Ping Hsu^1^, Ching-Ping Tseng^1,2,3^

^1^ Department of Laboratory Medicine, Chang Gung Memorial Hospital, Taoyuan County, Taiwan

^2^ Department of Medical Biotechnology and Laboratory Science, College of Medicine, Chang Gung University, Taoyuan County, Taiwan

^3^ Graduate Institute of Biomedical Sciences, College of Medicine, Chang Gung University, Taoyuan County, Taiwan

^4^ Clinical Informatics and Medical Statistics Research Center, College of Medicine, Chang Gung University, Taoyuan, Taiwan

^5^ Division of Allergy, Asthma, and Rheumatology, Department of Pediatrics, Chang Gung Memorial Hospital, Taoyuan, Taiwan

^6^ Department of Pediatrics, Division of Hematology/Oncology, Chang Gung Children’s Hospital, Chang Gung University, Taoyuan County, Taiwan

^†^ These authors contributed equally to the work.

**Address reprint requests to:** Ching-Ping Tseng

Ph.D. Professor

Department of Medical Biotechnology and Laboratory Science

Chang Gung University

Taoyuan City, 333, Taiwan

Tel.: +886-3-2118800 (ext. 5202)

Fax: +886-3-2118355

Email: [ctseng@mail.cgu.edu.tw](mailto:ctseng@mail.cgu.edu.tw)

| 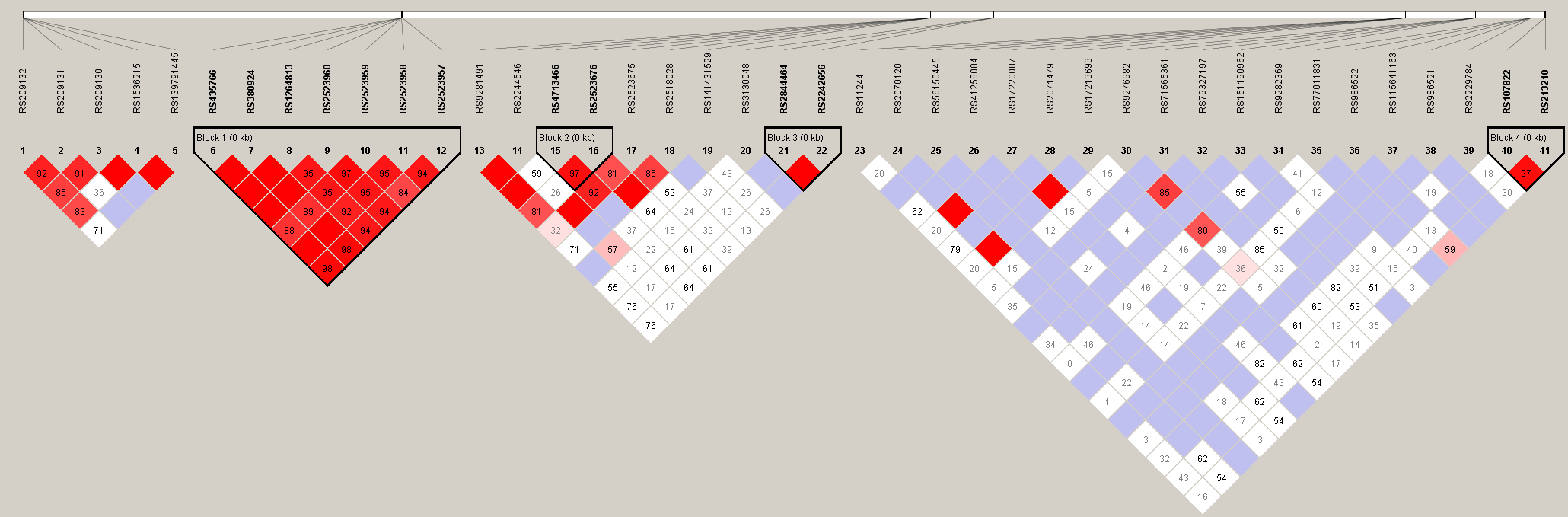 |
| --- |

**Figure S1:** Pair-wise linkage disequilibrium (LD) between the selected SNPs. All selected SNPs were analyzed through HaploView 4.2 Boxes with numbers refer to linkage disequilibrium (D’) between SNPs, boxes with no number in red color mean 100% linkage (D’ = 1) and in light purple color means 0% linkage. Colour legend: Red = high D’; white = low D’.
